# A Three-Year Longitudinal Follow-Up Study: Does Mild Cognitive Impairment Accelerate Age-Related Changes in Physical Function and Body Composition?

**DOI:** 10.7759/cureus.68605

**Published:** 2024-09-04

**Authors:** Hyuma Makizako, Shoma Akaida, Mana Tateishi, Daijo Shiratsuchi, Ryoji Kiyama, Takuro Kubozono, Toshihiro Takenaka, Mitsuru Ohishi

**Affiliations:** 1 Department of Physical Therapy, Kagoshima University, Kagoshima, JPN; 2 Graduate School of Health Sciences, Kagoshima University, Kagoshima, JPN; 3 Department of Epidemiology of Aging, National Center for Geriatrics and Gerontology, Obu, JPN; 4 Department of Cardiovascular Medicine and Hypertension, Graduate School of Medical and Dental Sciences, Kagoshima University, Kagoshima, JPN; 5 Department of Internal and Cardiovascular Medicine, Tarumizu Chuo Hospital, Tarumizu Municipal Medical Center, Tarumizu, JPN

**Keywords:** aging, longitudinal study, cognition, shrinking, muscle mass

## Abstract

Age-related declines in physical function, body composition, and cognitive function are interrelated. This prospective study aimed to examine the impact of mild cognitive impairment (MCI) on age-related changes in physical function and body composition among community-dwelling older adults. We analyzed data from 180 older adults (aged ≥70 years) who completed a longitudinal assessment of physical function and body composition in the community. Physical function included grip strength and time taken to walk 10 m at normal and maximum pace. Body composition assessments calculated the body mass index (BMI) and appendicular skeletal muscle index (ASMI) using bioelectrical impedance analysis at baseline and three-year follow-up assessments. MCI was defined as values below the age- and education-adjusted reference threshold in several tests, including memory, attention, executive function, and processing speed. Participants were divided into the MCI and non-MCI groups based on their MCI status at baseline. A two-way repeated-measures analysis of covariance (ANCOVA), adjusting for age and gender, was used to analyze the group (MCI and non-MCI) by time (baseline and three-year follow-up) interaction. Thirty participants (16.7%) had MCI at baseline. The repeated-measures ANCOVA indicated that no variables had significant group by time interactions. Stratified analyses by gender (repeated-measures ANCOVA, adjusted for age) confirmed a significant group by time interaction on BMI (F=5.63, p=0.02) and ASMI (F=6.33, p=0.01) among women. Older women with MCI may experience a greater impact of the acceleration of shrinking and age-related decline in muscle mass. The close associations of MCI with shrinking and muscle mass loss have important implications for targeting interventions among MCI women.

## Introduction

Age-related functional problems that are not severe diseases, such as mild cognitive impairment (MCI), frailty, and sarcopenia, may have greater impacts on healthcare systems serving an aging population. MCI is defined as a mental condition that lies between normal cognitive aging and early dementia [1].

The results of a recent mixed cohort study examining temporal trends indicated that older adults' cognitive functioning is improving [2]. However, many previous longitudinal studies have indicated that MCI has high conversion rates to dementia, ranging from 10% to 15% [3], and not only cognitive disorders but also physical problems, including physical frailty and sarcopenia, may be negative effects of MCI among community-dwelling older adults [4,5].

In addition to age-related cognitive decline, age-related changes in physical function and body composition have future adverse effects on health. Grip strength and walking speed are simple and easy to assess for physical function in the community setting. Several longitudinal studies found that grip strength [6] and walking speed [7] were good predictors for the incidence of disability among community-dwelling older adults. Therefore, grip strength and walking speed are useful markers for predicting poor health and included in assessment items for frailty and sarcopenia.

Body composition changes according to advancing age. Body mass index (BMI), calculated as weight divided by height (kg/m^2^), has been used to assess obesity. In late life, BMI declines with advancing age, and decreasing BMI is associated with higher mortality [8]. BMI is expressed using the full body weight; thus, there is a moderate decline compared with the appendicular skeletal muscle mass index (ASMI) [9], which is calculated by the appendicular skeletal muscle mass divided by height. The ASMI declines rapidly after age 65 years and is one of the essential markers of sarcopenia assessment.

Physical function indicators are also useful markers for the early detection of cognitive decline among community-dwelling older adults [10]. Although the association between cognitive status and physical function and body composition has been studied in community-dwelling older adults in cross-sectional studies, there have been few longitudinal studies [11]. Age-related declines in physical function, body composition, and cognitive function are interrelated. Additionally, there seem to be sex differences in functional declines of physical and cognitive status and changes in body composition [12]. This study aimed to investigate the effect of MCI on three-year longitudinal age-related changes in physical function and body composition among community-dwelling older adults.

## Materials and methods

Participants

**Figure 1:**
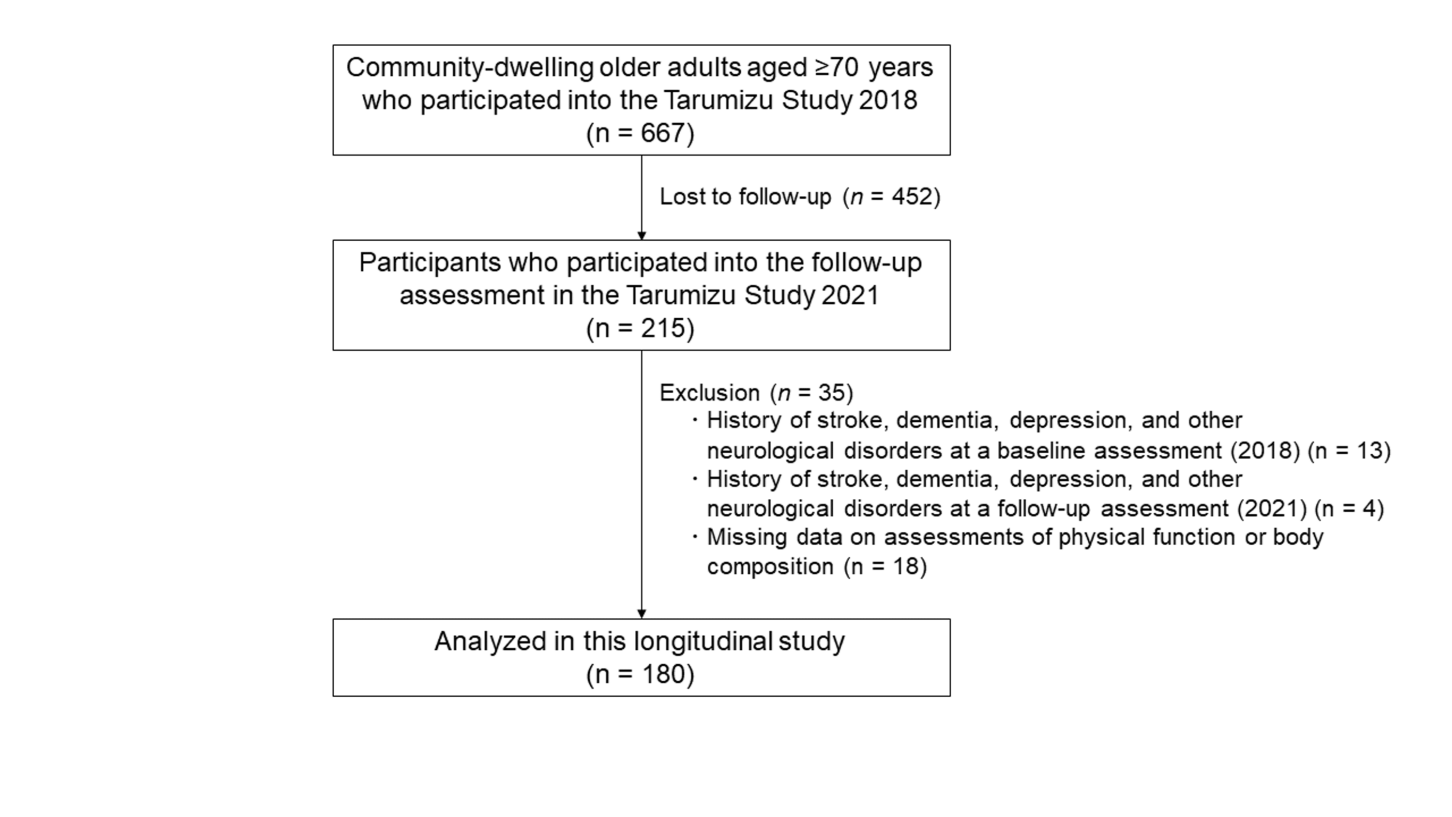
Participants' flow

The study participants were community-dwelling older adults aged ≥70 years who participated in the Tarumizu study and completed a longitudinal assessment of physical function and body composition. Older adults are defined as 65 years and older in most countries. However, the "rejuvenation" phenomenon is occurring among the new generation of Japanese older adults. Additionally, this longitudinal study applied a relatively short-term period (three years). Therefore, this study included older adults aged 70 years and older. The Tarumizu study is a health checkup survey of local residents, conducted in cooperation with Tarumizu Central Hospital, Kagoshima University (Faculty of Medicine), and Tarumizu City Hall. Participants in the Tarumizu study were selected from among citizens of Tarumizu City (a regional city in Kagoshima Prefecture) aged 40 years and older. This study used data from an administrative project conducted in Tarumizu City for all citizens aged 40 years and above. The 2018 Tarumizu study included 667 older adults aged 70 years or older, 215 of whom participated in the 2021 Tarumizu study. In the present study, the health check in the 2018 Tarumizu study was used as a baseline assessment, and a three-year follow-up assessment was held in 2021. Participants with a history of diagnosis of stroke (n=10), dementia (n=3), depression (n=2), and other brain disorders (n=2) in 2018 or 2021 were excluded. Participants with missing data for body composition measures (e.g., heart pacemakers) (n=5) and physical function measures (grip strength or walking speed) (n=13) who had unsafe conditions (e.g., systolic blood pressure ≥180 mmHg) in 2018 or 2021 were also excluded. Finally, data from 180 community-dwelling older adults (aged ≥70 years, mean age=75.3 years, 58.9% women) were analyzed (Figure 1). The Ethics Committee on Epidemiological and Its Related Studies of Kagoshima University, Sakuragaoka Campus (approval number: 170351), approved this study. Informed consent was obtained from all participants before their enrollment in the study.

Baseline assessment

Multidimensional cognitive function was assessed using the National Center for Geriatrics and Gerontology Functional Assessment Tool (NCGG-FAT) [13] for the baseline. The NCGG-FAT includes subtests in the following four areas: (1) memory (Word List Memory-I (immediate recognition) and Word List Memory-II (delayed recall)), (2) attention (tablet version of TMT-part A), (3) executive function (tablet version of TMT-part B), and (4) processing speed (tablet version of Digit Symbol Substitution Test). Memory tests comprised two computerized tasks: immediate recognition and delayed recall. Participants were instructed to memorize 10 words, each displayed for two seconds on the tablet PC. After that, participants were shown 30 words, including 10 target and 20 distracter words, and they were required to select the 10 target words immediately. This task was repeated for three trials. The average number of correct answers was recorded as a score ranging from 0 to 10. Participants were also asked to recall the 10 target words correctly after 20 minutes. The number of correctly recalled target words was scored, ranging from 0 to 10. The sum score of the two immediate recognition tasks and delayed recall was calculated. In the TMT-A task, participants were instructed to touch the target numbers (from 1 to 15) randomly displayed on the tablet panel in a sequence as rapidly as possible. In the TMT-B, participants were required to touch target numbers (e.g., 1-15) and letters. The time (seconds) needed to complete each task was recorded within a maximum of 90 seconds. In the Digit Symbol Substitution Test, nine pairs of numbers and symbols were shown in the upper part of the tablet display. A target symbol was shown in the center of the tablet panel, and selectable numbers were displayed at the bottom. Participants were asked to touch the number corresponding to the target symbol shown in the central part of the tablet display as rapidly as possible. The number of correct numbers within 90 seconds was recorded. In community-dwelling older adults, the NCGG-FAT has been shown to have high test-retest reliability, moderate to high criterion-related validity, and predictive validity [14]. MCI is defined as individuals who score below the criterion threshold (1.5 standard deviations), adjusted for age and education level on the four domain (memory, attention, executive function, and processing speed) subtests of the NCGG-FAT [14]. Participants were divided into the MCI and non-MCI groups based on their MCI status at baseline.

Outcomes

Physical function and body composition were assessed at baseline, and the three-year follow-up assessments were used as outcomes. Physical function included grip strength and time taken to walk 10 m at normal and maximum pace. Body composition assessments calculated participants' BMI and ASMI using bioelectrical impedance analysis at baseline and at the three-year follow-up assessments. Trained physical therapists assessed physical function, and walking time was measured automatically using infrared timing gates. All assessors were different from the person who analyzed the data.

Physical function

Grip Strength

Grip strength was assessed by the maximum grip strength (in kilograms) of the participant's dominant hand. The instrument used to evaluate grip strength was a Smedley-type hand-held dynamometer (GRIP-D; Takei Corporation, Niigata, Japan). The grip strength test was carried out once only.

Normal and Maximal Walking Time

Walking time was measured in seconds using an infrared timing gate (YW; Yagami Corporation, Nagoya, Japan). Participants were asked to walk a straight, flat, 10-m-long walking path at normal and maximum walking speeds, and infrared timing gates were placed at the 2-m point and at the end of the path. Normal and maximal walking time was measured each once.

Body composition

BMI

BMI was calculated using data from a body measurement including height (cm) and body weight (kg). Body weight was measured by using the InBody 430 (InBody Japan, Tokyo, Japan). BMI (kg/m^2^) was derived as the body weight in kilograms divided by the square of height in meters.

ASMI

Appendicular skeletal muscle mass (ASM) was assessed by a multifrequency bioelectrical impedance analysis using the InBody 430, which employs a four-pole, eight-point tactile electrode system to measure the impedance of the trunk, legs, and arms separately for each segment at three different frequencies (5, 50, and 250 kHz). Each participant's heel and forefoot were in contact with circular foot electrodes, and the surface of the hand electrodes was in contact with each of the five fingers. Participants were measured with their arms and legs in an extended position to avoid contact with other body parts during the measurement. ASM was derived as the sum of the muscle mass of the four limbs, and the ASM index (ASMI; kg/m^2^) was calculated.

Statistics

Data have been presented as mean±standard deviation (SD) for continuous variables and population (%) for categorical variables. The characteristics and baseline measurements of physical function and body composition were compared using the Student's t-tests and chi-squared tests between the MCI and non-MCI groups. A two-way repeated-measures analysis of covariance (ANCOVA), adjusted for age and gender, was used to analyze the groups (MCI and non-MCI) by time interaction (baseline and three-year follow-up). We also performed the two-way repeated-measures ANCOVA adjusted for age in men and women to test the main effects and group by time interaction. Data entry and analysis were performed using IBM SPSS Statistics for Windows, Version 25.0 (Released 2017; IBM Corp., Armonk, New York, United States). A p-value of <0.05 was considered statistically significant.

## Results

Thirty participants (16.7%) had MCI at baseline. Participants with MCI showed significant older age and poor physical performance (grip strength and normal walking speed) at the baseline assessment. There were no significant differences in body composition between the MCI and non-MCI groups (Table 1).

**Table 1:** Baseline characteristics BMI: body mass index; ASMI: appendicular skeletal muscle mass index

	Overall (n=180)	Non-MCI (n=150)	MCI (n=30)	p
Age, years	75.3±4.4	74.8±4.3	77.4±4.6	0.003
Women, n (%)	106 (58.9)	90 (60.0)	16 (53.3)	0.498
Education, years	11.7±2.5	11.9±2.5	11.1±2.1	0.102
Medication, n/day	2.9±2.8	2.9±2.8	3.0±2.5	0.828
Follow-up period, days	1140.1±70.3	1141.3±69.3	1134.3±76.1	0.617
Grip strength, kg	25.8±7.6	26.3±7.5	23.2±7.5	0.041
10-m walking time (normal), sec	7.8±1.4	7.7±1.3	8.0±1.7	0.044
10-m walking time (maximum), sec	6.1±1.0	6.0±1.0	6.4±1.3	0.054
BMI, kg/m^2^	23.2±3.1	23.1±3.2	22.6±2.7	0.429
ASMI, kg/m^2^	6.4±1.0	6.4±1.0	6.2±1.0	0.484

**Figure 2:**
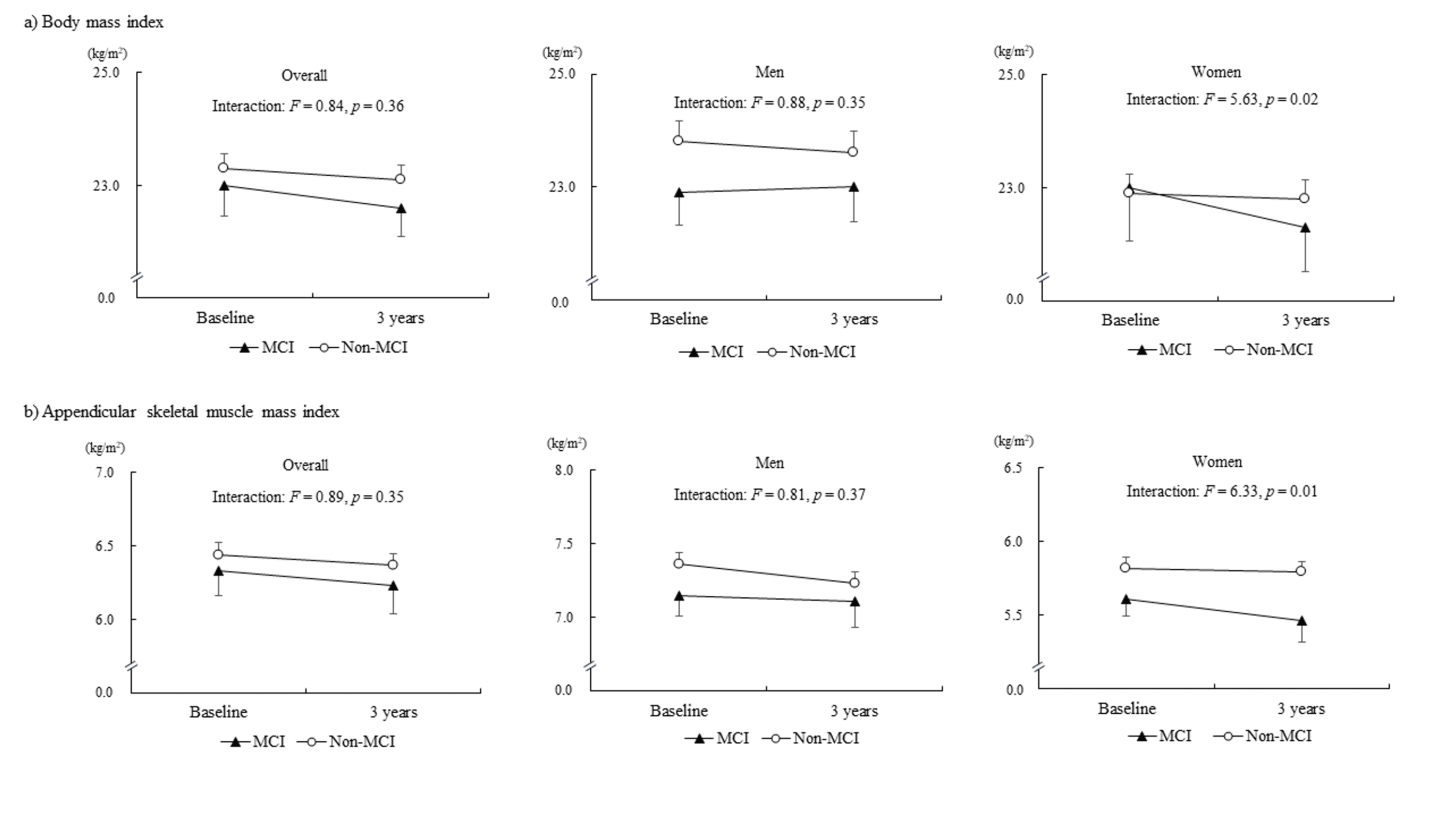
Changes and interactions of body composition

Table 2 presents the results of the repeated-measures ANCOVA adjusted for age and gender, and Table 3 presents conducting stratified analyses by gender. No variables had significant group by time interactions in the repeated-measures ANCOVA using overall data. The stratified analyses by gender (repeated-measures ANCOVA, adjusted for age) showed no statistically significant group by time interactions in physical performance and body composition in men. There were significant group by time interactions for BMI (F=5.63, p=0.02) and ASMI (F=6.33, p=0.01) among women. Figure 2 illustrates the changes and interactions in body composition between men and women.

**Table 2:** Mild cognitive impairment and age-related changes in physical function and body composition (repeated-measures ANCOVA) (overall, n=180) BMI: body mass index; ASMI: appendicular skeletal muscle mass index; SD: standard deviation; ANCOVA: analysis of covariance; ANOVA: analysis of variance Group: MCI/non-MCI groups; time: baseline/three-year follow-up ^a^ANCOVA modeling, using the three-year measures as the dependent and baseline measures and group (MCI or non-MCI) variables as independent variables. The beta coefficient for the group variable is presented in this table. ^b^Repeated-measures ANOVA modeled the measures as the dependent variable adjusted for age and gender and calculated the group (MCI and non-MCI) by time interactions (baseline and three-year follow-up).

	Baseline	Follow-up	ANCOVA regression coefficient^a^			Repeated-measures ANCOVA^b^ (group×time interaction)	
Mean±SD			Β	p	Adjusted R^2^	F	p
Physical function							
Grip strength	25.8±7.6	24.4±6.9	-0.02	0.98	0.81	1.00	0.32
Normal walking time	7.8±1.4	7.9±1.3	0.33	0.09	0.42	0.43	0.51
Maximal walking time	6.1±1.0	6.1±1.0	0.13	0.37	0.48	0.28	0.60
Body composition							
BMI	23.2±3.1	23.0±3.1	-0.20	0.38	0.87	0.84	0.36
ASMI	6.4±1.0	6.3±1.0	-0.04	0.55	0.90	0.89	0.35

**Table 3:** Mild cognitive impairment and age-related changes in physical function and body composition in men (n=74) and women (n=106) (repeated-measures ANCOVA) BMI: body mass index; ASMI: appendicular skeletal muscle mass index; SD: standard deviation; ANCOVA: analysis of covariance; ANOVA: analysis of variance Group: MCI/non-MCI groups; time: baseline/three-year follow-up ^a^ANCOVA modeling, using the three-year measures as the dependent and baseline measures and group (MCI or non-MCI) variables as independent variables. The beta coefficient for the group variable is presented in this table. ^b^Repeated-measures ANOVA modeled the measures as the dependent variable adjusted for age and calculated the group (MCI and non-MCI) by time interactions (baseline and three-year follow-up).

	Baseline	Follow-up	ANCOVA regression coefficient^a^			Repeated-measures ANCOVA^b^ (group×time interaction)		Baseline	Follow-up	ANCOVA regression coefficient^a^			Repeated-measures ANCOVA^b^ (group×time interaction)	
Mean±SD			Β	p	Adjusted R^2^	F	p			Β	p	Adjusted R^2^	F	p
Men (n=74)								Women (n=106)						
Physical function														
Grip strength	33.1±5.0	30.5±5.7	-0.08	0.95	0.49	0.70	0.41	33.1±5.0	30.5±5.7	-0.36	0.57	0.67	0.47	0.50
Normal walking time	8.0±1.4	7.9±1.2	0.14	0.61	0.44	0.56	0.46	8.0±1.4	7.9±1.2	0.52	0.06	0.43	2.20	0.14
Maximal walking time	5.9±1.0	5.8±0.9	0.13	0.53	0.37	0.73	0.40	5.9±1.0	5.8±0.9	0.21	0.25	0.56	0.00	0.97
Body composition														
BMI	23.6±2.8	23.4±2.9	0.29	0.46	0.81	0.88	0.35	23.6±2.8	23.4±2.9	-0.60	0.03	0.90	5.63	0.02
ASMI	7.3±0.6	7.2±0.7	0.08	0.46	0.71	0.81	0.37	7.3±0.6	7.2±0.7	-0.13	0.07	0.84	6.33	0.01

## Discussion

This longitudinal study with a three-year follow-up period indicated that there were no significant effects of MCI in age-related changes in physical function and body composition. However, the results of stratified analyses by gender suggested that older women with MCI may experience a greater impact on the acceleration of shrinking and age-related decline in muscle mass.

Previous observational studies showed longitudinal changes in physical function in older adults with MCI. Having MCI or dementia is associated with greater physical decline compared to older people with normal cognition [15]. Older adults with MCI converted to dementia more rapidly than those without physical functional impairment [16]. Older adults with MCI may develop Alzheimer's disease (AD) in approximately 3.5 years, and both cognitive and physical function may decline gradually after AD onset [17]. In addition, physical functional limitations may suggest early signs of cognitive deficits [18]. Two-thirds of MCI were physically frail or pre-frail, mostly due to low lean muscle mass, slow gait speed, or balance and gait impairment [19]. There are close interactions between age-related declines in cognitive and physical function. Thus, targeting interventions for both functions will be needed to prevent dementia and disability among older individuals.

This longitudinal study indicated that MCI leads to the acceleration of shrinking and age-related decline in muscle mass among older women. Not only physical function but also body composition changes with advancing age. There is a possibility of a difference in the age-related slope patterns among the parameters of body compositions. In particular, the age-related decrease in ASMI is more striking than that of BMI [9]. Those striking slopes are observed earlier in women than in men [9]. Cognitive status may be an important factor for the acceleration of loss of muscle mass, and direct and indirect relationships between cognitive status and acceleration of loss of muscle mass should be considered. For instance, cognitive deficits are associated with physical inactivity in older adults [20], and physical inactivity may accelerate the age-related decline in body composition [21]. Additionally, undesirable lifestyles, such as poor diet patterns [22] and sleep problems [23], coupled with cognitive deficits would affect shrinking among older adults.

On the other hand, previous studies have reported the negative impacts of shrinking among older adults' future health outcomes, including cognitive function. Lower baseline BMI has been associated with a more rapid cognitive decline in MCI [24]. In addition, a higher BMI has been associated with a lower risk of dementia, and being underweight was associated with a higher risk of dementia [25]. BMI has been associated with an increased risk for dementia; however, the "BMI paradox in dementia" requires further discussion [26]. Being overweight in mid-life has been associated with an increased risk of dementia in later life, whereas being overweight in later life has been linked to reduced dementia risk [27]. As our longitudinal study indicated, MCI accelerated shrinking and loss of muscle mass among older women. Thus, the effects of cognitive status should be considered to reduce the risk of adverse health impacts due to shrinking in late life.

This study found no significant relationships between MCI and age-related changes in physical function and body composition in older men. Sex differences in the association between sarcopenia and MCI have been found in previous studies [28], with significant associations between sarcopenia and MCI in older women but no significant relationship in older men [28]. The impacts of cognitive status on muscle mass loss with advancing age may be greater in older women. The mediating factors between cognition and muscle mass loss will need to be clarified to understand these sex differences. For instance, cognitive status affects the levels of activities of daily living (ADL). Older adults with MCI, including during the prodromal stage, show steeper rates of decline in complex ADL and daily functioning than those with normal cognition [29]. Older adults' interest in, participation in, and satisfaction with instrumental ADLs and leisure time activities may differ by sex [30]. Activities with physical and cognitive stimulation may mediate the relationships between cognitive status and age-related changes in physical function and body composition.

Limitations

Several limitations in this study should be noted. The three-year follow-up assessment conducted in the 2021 Tarumizu study had a limited sample due to the COVID-19 pandemic situation. Consequently, the follow-up rate in this study was low (approximately 30%). In addition, relatively healthy older adults may have participated in both the baseline and follow-up assessments. Age-related changes in this study may be underestimated due to the survival effects. Therefore, we should carefully consider whether this sample size is justifiable. In this study, associated factors with age-related changes in physical function and body composition, such as physical activity and nutrition, were not considered. Older adults decreased their physical activity and might have a poorer nutritional status under the COVID-19 pandemic situation. These temporary lifestyle changes may have led to accelerated declines in physical function and body composition. Although MCI affects age-related changes in body composition, shrinking, and muscle loss (e.g., sarcopenia), it may also accelerate cognitive deficits. These interactive associations should be considered to assess the functional trajectory and plan preventive strategies.

## Conclusions

No significant effects of MCI in age-related changes in physical function and body composition were confirmed. However, older women with MCI may experience a greater impact of the acceleration of shrinking and age-related decline in muscle mass. The close associations of MCI with shrinking, including muscle mass loss, have important implications for targeting interventions among MCI women to prevent adverse health effects, such as sarcopenia and disability.
